# The Relationship Between Face-Based First Impressions and Perceptions of Purity and Compared to Other Moral Violations

**DOI:** 10.3390/bs14121205

**Published:** 2024-12-16

**Authors:** Kate McCulloch, Yoshi Steele, Ana I. Gheorghiu

**Affiliations:** 1 Department of Psychology, University of Suffolk, Ipswich IP4 1QJ, UK; 2 School of Psychology Sport and Health Sciences, University of Portsmouth, Portsmouth PO1 2UP, UK; yoshi.steele@port.ac.uk (Y.S.);

**Keywords:** face perception, morality, purity, moral foundations, social judgements

## Abstract

A trait labelled as “morality” has been argued to be perceived and prioritised during first impressions of faces; however, immorality is not a homogenous concept. Violations of purity are frequently distinguished from other violations via distinct behavioural and emotional patterns, arguably stemming from physical disgust, sexual content, or “weirdness” impure scenarios. In the current research, participants were asked to rate unfamiliar faces based on social traits and their likelihood of engaging in immoral or nonmoral behaviours. Across two studies, perceived engagement in most autonomy and purity moral violations but also the non-moral sexual and “weird” acts was predicted by lower facial morality. There was also a distinction wherein most purity violations and physical disgust were more associated with male gender, and most autonomy violations with ratings of high dominance. The scenarios also differed within categories, such as sexually impure scenarios and disgust associated with unattractive faces; while behaving “weirdly” and certain other purity violations were associated with low dominance. Taken together, our results suggest faces seemingly low on the trait labelled “morality” are perceived as more likely to engage in most immoral behaviours, but also in other socially relevant, nonmoral behaviours. Social judgements are also not homogenous within or between theory-based categories of moral violations.

## 1. Introduction

It is common wisdom that one should “never judge a book by its cover”, yet our initial perceptions of an individual’s facial appearance seem key to forming our deeper judgements about them. In addition to superficial judgements such as facial attractiveness, people judge socially relevant characteristics such as how competent, sociable, trustworthy, and dominant a person may be based solely on their facial appearance [1,2,3]. These face-based first impressions of social traits have been shown to have important consequences for real-life outcomes; for example, people use a political candidate’s perceived competence to influence their voting behaviour [4], a defendant perceived as having a stereotypically Black appearance leads to negative criminal justice decisions [5,6,7], a military leader’s facial dominance influences judgements of their efficiency and skill [8,9], and a speaker’s attractiveness to decide whether they are a “good” and “interesting” scientist [2]. Interestingly, previous research has also shown that people not only make these judgements but are also surprisingly accurate and in high agreement when inferring social traits from face-based first impressions [10]. Using “thin slices” of exposure to images or short videos of strangers, there is evidence of accurate judgements of personality [8,9,10,11,12,13,14], dishonesty [15], violence [16], sexual orientation [17,18], and social status [19,20,21]. Considering humans perceive numerous social traits from appearance alone with seemingly remarkable accuracy, the confidence we have in these judgements may have important implications for our real-world social judgements.

One such social judgment concerning someone’s perceived morality has been found to play a fundamental role in assessing who may be beneficial or harmful to individuals and groups [22,23]. This moral perception is conceptualised within impression formation theories as involving either two or three core dimensions. The two-dimensional approaches typically assess faces on a dimension highlighting someone’s intentions (usually trustworthiness or warmth, which includes elements of trustworthiness and morality) and a dimension reflecting perceived capability to enact these intentions (usually coined competence, dominance or power) [1,24]. These judgments are thought to have evolved as mechanisms for evaluating potential allies or threats. The three-factor model of social perception extends these earlier frameworks by proposing that competence, sociability, and morality are the core social traits used in forming first impressions, with morality being the most critical dimension [2,3,25]. Across all models, morality is generally defined as the apparent trustworthiness and honesty of the target, but they differ on whether this is a separate judgment from warmth or not.

Impression formation research has highlighted that judgements about the morality of a person’s appearance are prioritised due to their pivotal role in determining intentions and assessing threats [22], with those judged as immoral being considered a safety risk to the individual, the group, or the reputation of the group from association with an immoral actor. The importance of morality to social life is also supported by research outside of impression formation theory, such as in evolutionary research, where morality is also deeply tied to cooperation. Specifically, it has been argued that morality evolved to support social cohesion [26,27], with cooperative behaviours framed as morally positive [26]. It also should be noted that the specific negative judgements about a person’s perceived immorality lead to negative social consequences for that person, who is more likely to be rejected from social groups [28]. The importance of first impressions of immorality is further highlighted by the neural mechanisms involved. Perception of trustworthiness is processed more quickly than sociability or competence and relies on the amygdala, which is a brain structure implicated in processing other threatening stimuli [23,29,30]. It is argued that perceived morality informs us of a stranger’s intentions, which is particularly beneficial to deciding whether we wish to approach them or not [25]. Other social dimensions such as competence (i.e., a stranger’s ability to carry out their intentions) and sociability (i.e., a stranger’s support network in carrying out their intentions) are secondary and only relevant once we have determined the valence of their intentions [25]. Due to the potential reputational risks of associating with humans who may behave immorally and the risks of those people harming individuals or the group with their behaviours, there is a clear motivation to prioritise social judgements of immorality during first impressions.

However, within impression formation research, morality has been treated as a single construct associated with low-level social harm, such as cheating and lying, when it is, in fact, diverse, with different categories of moral violations eliciting distinct emotional and behavioural responses [31,32,33,34]. Previous impression formation research has focused on one category of immoral behaviours, often called autonomy violations [22,23,35,36]. Autonomy violations focus on individual rights and protection from harm but are distinct from other violations, including “divinity”, also called “purity”, which focuses more on spiritual traditions, natural order, and pollution of the body or soul [31,32,33,37,38,39,40,41,42,43]. Violations of purity or divinity are widely used and have been broadly defined as inhuman, corrupting, polluting acts, including sexual violations, such as incest, or food violations, such as cannibalism [44]. However, it has been noted there are important inconsistencies in the approach to defining purity this broadly [37]. Whether facial morality is also associated with other forms of moral violations, such as purity violations, has not been investigated.

Moral Foundations Theory posits that moral violations fall into distinct categories, each tied to evolved social functions and emotional responses: purity/degradation avoids contamination and upholds sanctity, driven by disgust; care/harm promotes kindness and protects the vulnerable, rooted in empathy; fairness/cheating ensures cooperation and equity, evoking anger at dishonesty; loyalty/betrayal fosters group cohesion and punishes defection, tied to pride and belonging; and authority/subversion upholds hierarchies and traditions to maintain order, associated with respect and fear of instability [31,38,45,46]. Research highlights a notable distinction between purity violations, which elicit disgust, and autonomy violations (related to care and fairness foundations), which elicit anger [41,47,48,49,50,51]. Thus, the current research focuses on comparing these two categories of moral violations, as they may differ in the social judgments they elicit. However, while some research would focus on the distinction between these two categories, an alternative framework, the Affective Harm Account of moral judgment, emphasises the similarity of the harm caused by purity and autonomy violations [52]. This account proposes that moral wrongness arises from harm to emotional or experiential states. Purity violations elicit disgust or psychological harm, while autonomy violations involve physical or emotional harm. Both are viewed as morally wrong because they inflict affective harm. From this perspective, purity and autonomy violations are both seen as threats to individuals or groups, which would support the prioritisation of similar moral judgments in first impressions for both forms of violation [22,23,35,53].

Purity violations often involve extreme behaviours that are considered distinct from other moral infractions, most often due to a consistent disgust response and perceptions of greater social harm to the violator [35,49,50,51,52]. In support of the Affective Harm Model, one might argue that violations such as cheating, stealing, and lying should be prioritised in impression formation as they directly harm others and undermine trust. However, purity violations may be considered more socially harmful. Purity violations have been associated with deeper flaws in moral character and are frequently regarded as more socially damaging than autonomy violations [39,49,50,54,55,56,57]. Furthermore, research focused on the disgust response to immorality has also noted that disgust evolved to protect us from both physical and social parasites [58], and it forms part of a Behavioural Immune System which is primed to quickly perceive potentially harmful stimuli (including other humans) and encourage protective, avoidant behaviours in response [59,60]. Due to the importance of protecting the person from social or physical disease, this disgust response also relies on harsh, immediate judgements of people, which may be inaccurate [61], similar to other first impressions. Disgust is also argued to encourage socially relevant avoidant behaviours [62,63,64,65], such as ostracising or gossiping about the target. Anger, the emotion more associated with violations of autonomy, is thought to encourage an approach in the form of verbal or physical aggression directed at the target [34,62,63,64]. However, it has been suggested this emotional distinction and associated social behaviour does not map directly onto moral categories and, instead, can be related to the level of personal risk: the same moral violation can become more anger-inducing when targeting you and more disgust-inducing when targeting someone else [65,66]. Whether mapping these emotions to the category of violation or the target of the violation, again, the motivation appears to be to protect individuals and the group from immoral behaviour, which has been argued to be the function of appearance-based judgements of morality [22,23,35,53]. In either case, therefore, it would be socially protective to form early impressions of distrust in someone who may violate impurity, not just autonomy.

Overall, we can see that there is currently agreement that perceptions of facial “morality” are prioritised and associated with expectations the person will commit certain harmful, immoral behaviours [22,23,35,36]. However, impurity is a distinct category of immoral behaviour, which is still of social consequence to the reputation of the group and potentially risky to the individual. The importance of not limiting impression formation research to autonomy violations can be seen in the negative social consequences of being judged low in morality [28] and the evidence that “disgusting” purity violations are even more socially damaging than other forms of moral violation [39,49,50,54,55,56,57]. However, we do not yet know whether the pre-established, consistent initial impression of dishonesty and untrustworthiness is just as likely to be associated with that actor committing an autonomy violation, like stealing, as a purity violation, such as incest or cannibalism. It is also currently unclear which other facial traits commonly used in impression formation research [2,3], such as gender, age, ethnicity, and attractiveness, may be associated with assumptions about engaging in immoral behaviours.

We, therefore, aim to explore social judgements about who seems likely to commit specific categories of immoral acts (i.e., purity and autonomy violations). Our findings will contribute to our understanding of whether there are differing expectations of the superficial facial traits possessed by an “impure” actor. This will help explain in further detail what stereotypes people hold about the traits associated with different forms of immorality, which may impact their first impressions of these individuals. We expect that social variables such as competence and sociability will not show a consistent pattern with immoral scenarios, as their relevance will be dependent on perceptions of the competence and social network required to engage in the scenario. While it is possible facial judgements will vary like the emotional and behavioural responses to purity violations [31,38], due to the particular social relevance and evidence of social harm for immorality (including impurity [52]), we predict that faces judged as immoral (i.e., low in trustworthiness and honesty) will be considered more likely to engage in both purity and autonomy violations. We do not have any other specific predictions for the other face-based variables: age, gender, ethnicity, and attractiveness.

## 2. Ethical Approval and Informed Consent

All studies were reviewed by the University of Portsmouth Science and Health Ethics Committee (Study 1: SFEC 2020-031; Studies 2 and 3: SHFEC 2023-058) and are in accordance with BPS ethical guidelines and regulations. Participants provided online informed consent and were able to leave the study at any point without providing a reason.

## 3. Study 1

In Study 1, we aim to investigate which face-based social traits predict being perceived as being likely to engage in purity and autonomy violations. We measured perceptions of eight face-based social traits (competence, capability, likeability, friendliness, honesty, trustworthiness, dominance, and masculinity) and used confirmatory factor analysis to verify what factors these traits load on to (e.g., competence, morality, sociability [22,25] or competence/dominance/power and warmth/trustworthiness [1,24]). As criteria, we measured the perceived likelihood of engaging in purity and autonomy violations using scenarios adapted from the previous literature. Additionally, we used pre-existing measures of attractiveness, age, gender and ethnicity for our stimuli [67], and we controlled for individual differences in participants’ anger and disgust towards the scenarios they were presented with. We focus our predictions on the most relevant social trait: morality; as such, we hypothesise that low perceived morality will predict the likelihood of engaging in both purity and autonomy violations.

### 3.1. Method

#### 3.1.1. Sample Size

The minimum sample size is based on previous impression formation research, recommending a minimum of 16 to ideally 25 participants per social judgement to yield acceptable reliability [1,2,3]. We collected 350 participants, equivalent to 25 participants, for each social judgement. Out of the initial sample, 24 entries were excluded for being incomplete responses (*n* = 9), being duplicate IPs (*n* = 2), reporting having started the survey more than once (*n* = 6), reporting issues with viewing the photos (*n* = 1) or having zero variance in their data (*n* = 6), resulting in a final sample of 326 participants.

#### 3.1.2. Participants

The final sample of 326 participants (201 females, 124 males, and 1 other) were recruited through Prolific Academic; each was paid £3 for completing the study. Ages ranged from 18 to 73 (M_age_ = 34.29, SD_age_ = 11.68). Of the 326 participants, 321 self-reported English as their native language, with the remaining 5 reporting speaking English fluently.

#### 3.1.3. Design and Materials

Participants were asked to judge all stimuli on a single dimension; they were either asked to rate faces on one of eight social traits (competence, capability, likeability, friendliness, honesty, trustworthiness, dominance, and masculinity) or one of six purity violation and autonomy violation scenarios (Table 1), resulting in 14 conditions. The between-subjects design is preferred in order to avoid carry-over effects [68,69].

As stimuli, we used 102 neutral, portrait-style photos of individuals from the Face Research Lab London Set [67]. This face database provides perceived attractiveness ratings for all the faces which we used in our analysis.

The three purity and three autonomy scenarios were adapted from previous research [39,70,71,72], edited so those committing the violations are nameless and converting any pronouns to be gender-neutral, as the stimuli encompassed photos of both men and women (Table 1).

#### 3.1.4. Procedure

Participants were randomly allocated to one of the 14 conditions (i.e., one of eight social traits or one of six scenarios), where they rated all 102 faces on their perceived trait/likelihood of engaging in the scenario; each face-rating combination represented a trial. In each trial, participants were given a prompt at the top of the screen reminding them of the task (e.g., “How COMPETENT is this person?” for social trait ratings, or “How likely do you believe the person in the image is to engage in the following scenario:”, followed by the purity or autonomy violation scenario), with the photo and response scale directly below. Responses were made using the keyboard on a Likert scale from 1—Not at all to 9—Extremely. The order in which the photos were presented was randomised for each participant.

After the face-rating task, participants in the six purity/autonomy scenario conditions were asked to rate how disgusted and angry they felt after reading the scenario (see Table 1 for scenarios); both responses were made on a Likert scale from 1—Not at all to 9—Extremely. Finally, participants provided demographic information on their gender, age, ethnicity, and level of education, as well as self-reporting starting the survey more than once or encountering issues with the photo display. The study was run using the software package Testable [73]. Participants could complete the study on any personal device with a keyboard (due to the response scale) and in their own time.

### 3.2. Results

#### 3.2.1. Data Preparation

Average attractiveness ratings for each face were computed based on the raw attractiveness scores available from the Face Research Lab London Set [67]. Face and participant gender (male = 0, female = 1) and face ethnicity (White = 0, any other ethnicities = 1) were re-coded in line with previous research from Gheorghiu et al. [2,3].

#### 3.2.2. Internal Reliability

Cronbach’s Alpha was used to assess the internal reliability of the ratings for all eight facial traits and six purity and autonomy scenarios.

Reliability was acceptable overall (i.e., Cronbach’s alpha was above 0.7; see Supplementary Table S1–1), with two exceptions: the “train” autonomy scenario (α = 0.22) and the “corruption” autonomy scenario (α = 0.63). Since the “train” scenario reliability is so low, we have decided to exclude this scenario from any further analyses—it seems people disagree strongly on this topic, which may be an artefact of the trolley dilemma’s popularity among the general public. We include the “corruption” scenario in our future analyses, with the acknowledgement that reliability is below the preferred threshold of α = 0.7.

#### 3.2.3. Confirmatory Factor Analysis

A confirmatory factor analysis (CFA) was conducted to verify whether the three-factor model of social judgement (competence, sociability and morality [25]) is a better fit for this data than the classic two-factor model of social judgement (competence and warmth [24]), and to assess whether dominance and masculinity should be included under the “competence/power” dimension [1] or be treated as standalone factors.

Based on the global indices of fit, the three-factor model with dominance and masculinity as separate factors was the best fit (SRMR = 0.033, RMSEA = 0.123, CFI = 0.973, TLI = 0.936), further supported by chi-square tests for the difference in model fit (see SI for a full breakdown of the analysis). Given the better fit of the three-factor model with dominance and masculinity as separate factors and the theoretical relevance of treating morality and sociability as separate dimensions in a study investigating morality [25], we will use the above model for this study’s analyses. As such, composite traits were calculated by averaging across the corresponding items, according to the CFA.

#### 3.2.4. Mixed Effects Models

Both the trait and scenario ratings data and participant-level data were collated and analysed to assess the impact of the face-based traits (competence, morality, sociability, collapsed from the collected ratings as a result of the CFA, dominance, masculinity and attractiveness; the latter were collected from Face Research Lab London Set [67]), demographic characteristics of the face (age, gender and ethnicity; collected from Face Research Lab London Set [67]) and participant demographic characteristics (age, gender, self-reported anger and disgust towards the scenario) on to the criterion variables (i.e., ratings of the likelihood of engaging into each of the purity and autonomy scenarios). Zero-order Pearson correlations between the predictors revealed that face gender and masculinity were strongly correlated (*r* = −0.98, *p* < 0.001). To avoid multicollinearity issues with the gender variable, we did not include masculinity as a fixed effect in the mixed-effects analysis (see Supplementary Materials, Tables S3 and S4 for full justification).

Face-level variables were standardised at the level of the face, and participant-level variables (i.e., participant demographic characteristics) were standardised at the participant level for each scenario.

Using lme4 [73] and Satterthwaite’s approximation (lmerTest [74]), we analysed the data with a mixed effects model including fixed effects of the face’s age, gender, ethnicity, attractiveness, competence, sociability, morality and dominance, and the participant’s age, gender, anger and disgust, and the following random effects: random intercepts for each participant and each face, as well as random uncorrelated slopes for the by-age, by-gender, by-ethnicity, by-attractiveness, by-discipline, by-competence, by-sociability, by-morality, by-dominance and by-masculinity effect of participant, and for the by-participant age, by-participant gender, by-participant anger and by-participant disgust effect of face. This model was fitted for each of the three purity and two autonomy scenario ratings with the “bobyqa” optimiser (Supplementary Materials Tables S5–S9).

**Figure 1:**
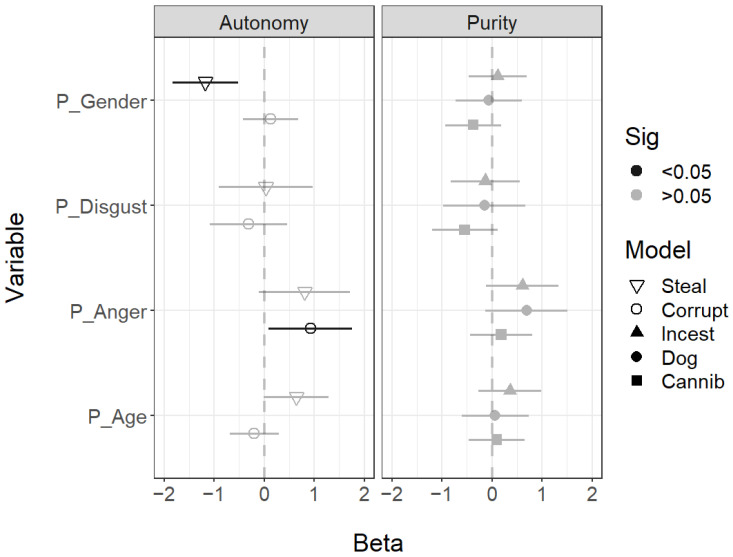
Regression coefficients for the participant variables. All predictors were standardised. Error bars show 95% confidence intervals; coefficients with CIs that exclude zero are highlighted in black. P_Disgust, participant disgust; P_Anger, participant anger; P_Age, participant age; and P_Gender, participant gender (1 = female).

The participant characteristics were mostly not associated with different ratings for all scenarios. The only differences were male participants viewed all faces as more likely to steal, while participants who felt more anger after reading the scenario perceived all individuals as more likely to be corrupt (Figure 1).

**Figure 2:**
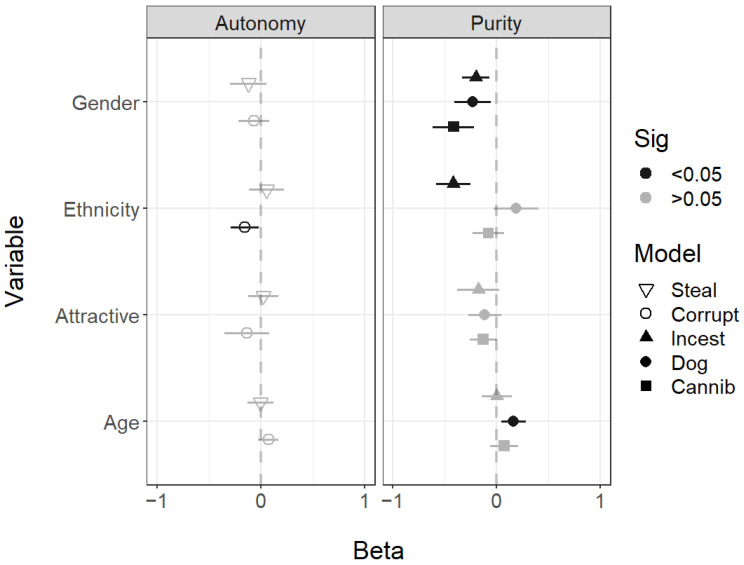
Regression coefficients for the face characteristic variables (Gender, Female = 1; Ethnicity, White = 1). All predictors were standardised. Error bars show 95% confidence intervals; coefficients with CIs that exclude zero are highlighted in black.

The facial traits of gender, ethnicity, attractiveness, and age were differently associated with the scenarios. The presented faces of white ethnicity were thought more likely to commit incest and be corrupt, while those older-looking faces were perceived as more likely to eat dog meat. A pattern did emerge for gender, which did seem to separate purity and autonomy violations—male faces were seen as more likely to engage in all impure scenarios. Face gender did not predict engagement in autonomy violations (Figure 2).

**Figure 3:**
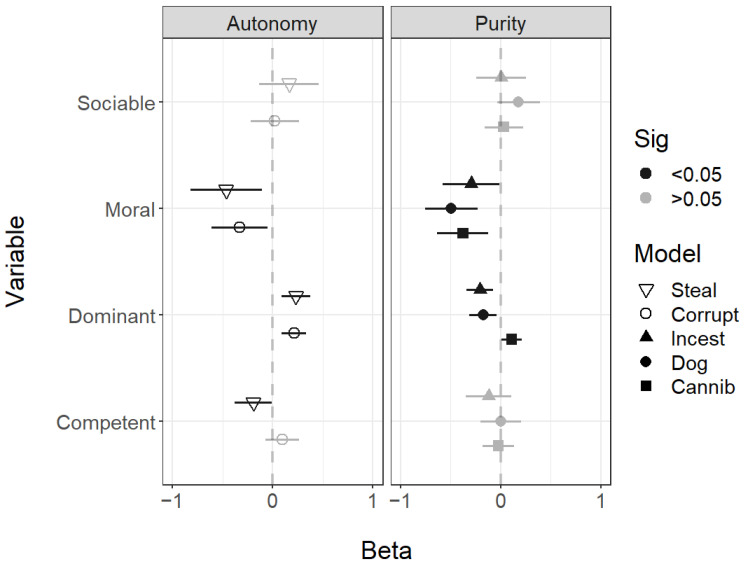
Regression coefficients for the face trait variables. All predictors were standardised. Error bars show 95% confidence intervals; coefficients with CIs that exclude zero are highlighted in black.

For the rated social facial traits of sociability, morality, competence, and dominance, the most consistent finding was that all moral violations were associated with faces low in morality. For dominance, those looking less dominant were perceived as more likely to commit incest and consume dog meat, while those looking more dominant were associated with both autonomy violations and more likely to commit cannibalism purity violations. Sociability was not associated with any of the scenarios, but faces with low competence were seen as more likely to steal (Figure 3).

Finally, to allow a more direct comparison to the results of Study 2, we subsequently re-ran the mixed effects models above without including fixed and random effects for participant anger and participant disgust. This did not result in any noteworthy changes to the direction or significance of the fixed effects, reinforcing our decision not to include measures of participant anger and participant disgust in Study 2, as it seems these individual differences in the emotion felt do not alter which characteristics emerge as predictors of engaging in the scenario.

### 3.3. Discussion

In Study 1, we found different characteristics were associated with the perceived likelihood of engaging in three purity violations and two autonomy violations. All moral violations—both purity and autonomy—were associated with lower perceived facial morality (i.e., lower perceived trustworthiness and honesty). However, there was more distinction in other facial traits. For example, for purity violations, the male gender was associated with all three scenarios, while autonomy violations were not associated with either gender. Lower facial dominance was associated with incest and eating the dead pet dog purity scenarios, but both autonomy violations and cannibalism purity violations were associated with higher facial dominance. Additionally, stealing was associated with lower competence, eating a pet dog was associated with older age, and both corruption and incest were associated with white ethnicity. Taken together, there was evidence of an emerging trend of purity violations being associated with faces that were immoral, low dominance, and male, whereas autonomy violations were associated with immoral, high-dominance faces of both genders. This suggests some distinction in the facial traits assumed to be connected to autonomy compared to purity violations.

However, these trends could be due to other characteristics in the scenarios than their purity or autonomy categorisation. It has been suggested that the distinct response to impurity, specifically feeling disgust rather than anger, is more related to the “weirdness” or novelty of the stimuli presented [75], the disease risk inherent in the scenario [76,77,78], and is inextricably linked to the sexual content often present in scenarios, including quite commonplace (although sometimes non-traditional or non-heteronormative) sexual behaviours [79,80]. As such, it is worth investigating a wider range of scenarios which vary in their morality, sexual content, disgust, and weirdness. We also wanted to investigate whether the other two factors of the three-factor model—competence and sociability—appeared to be associated with scenarios showing those traits.

## 4. Study 2

In Study 2, our overall aim is to investigate whether face-based social judgements (competence, morality, sociability), facial characteristics (attractiveness), and facial demographics (age, gender, and ethnicity) are differently associated with autonomy violations (weird and nonweird), purity violations (weird and nonweird, sexual and nonsexual), nonmoral scenarios containing single elements associated with impurity (sexual, weird, and disgusting), and scenarios displaying the other two traits from the three-factor model (incompetent and unsociable). To facilitate this, we begin in Study 2a with a survey to create stimuli. We then will use these stimuli for Study 2b, which follows the methodology of Study 1. Based on Study 1, we hypothesise that faces that are low morality, male, and low dominance will be considered more likely to engage in scenarios that are immoral, weird, and impure. We also hypothesise that low morality and high dominance faces will be considered more likely to engage in scenarios that are immoral autonomy violations. Finally, we aim to look further at the other facets of the three-factor model of face perception by creating unsociable and incompetent scenarios. Therefore, we also hypothesise that faces low in competence will be seen as more likely to behave incompetently and faces low in sociability will be considered more likely to behave unsociably.

## 5. Study 2a

In Study 2a, we aim to create additional immoral scenarios which vary in their sexual content and “weirdness”, as well as creating comparisons which do not violate morality. First, we wanted to vary both purity and autonomy violations in their weirdness as well as create purity violations which focus on both food and sexual violations. We also aimed to create some nonmoral comparison scenarios, which also vary in their sexual content, disgust, and weirdness, as well as creating scenarios that tap into the other two facets of the three-factor model: sociability and competence. To do this, we created 40 scenarios inspired by previous research and definitions of the concepts [31,32,33,37,38,39,40,41,42,43,44,49,50,70,71] and investigated whether they varied on their ratings of general immorality, purity, autonomy, weirdness, physical disgust, competence, and sociability.

### 5.1. Method

#### 5.1.1. Participants

Aiming for a minimum of 16 participants for a reliable consensus [2,3], we recruited 31 participants (21 females, 10 males) through Prolific Academic. Each was paid £4 for completing the study. Ages ranged from 24 to 62 (M_age_ = 36.48, SD_age_ = 8.47). Of the 31 participants, 29 participants self-reported English as their native language, while 1 reported Swahili and another Korean.

#### 5.1.2. Procedure

The study was an online survey using Qualtrics [81], in which participants rated the 40 scenarios. Participants were asked to provide basic demographic information (i.e., age, gender, ethnicity, and native language). Participants were then shown all 40 scenarios, one at a time and in a randomised order. After reading each scenario, participants were asked to rate the following aspects: negativity and immorality (3 questions: “How [NEGATIVE-TO-POSITIVE]/[MORAL OR IMMORAL] is this scenario?”; “How much have you considered MORALITY when reading this scenario?”), purity violation (3 questions: “How [ANIMALISTIC]/[DEGRADING]/[SINFUL] is this scenario?”), autonomy violation (2 questions: “How much have you considered [HUMAN FREEDOMS/RIGHTS]/[HARM] when reading this scenario?”), weirdness (3 questions: “How much have you considered WEIRDNESS when reading this scenario?”; “How [UNUSUAL]/[PECULIAR] is this scenario?”), physical disgust (2 questions: “How much have you considered [DISEASE]/[DISGUST] when reading this scenario?”), competence (1 question: “How much have you considered COMPETENCE when reading this scenario?”) and sociability (1 question: “How much have you considered SOCIABILITY when reading this scenario?”). All responses were measured on a Likert scale of 1—Not at all to 7—Very much so/Extremely (as applicable; or 1—Negative to 7—Positive for the first measure) due to a large number of questions, and to avoid confusion, the order of the measures was fixed for all participants.

### 5.2. Results and Discussion

The responses for the “negative-to-positive” item were reverse coded so that higher numbers indicate more negativity. All multiple-item measures showed acceptable reliability between items: negativity/immorality (3 items; α = 0.713), purity (3 items; α = 0.739), autonomy (2 items; α = 0.621), weirdness (3 items, α = 0.939), and physical disgust (2 items, α = 0.673). Both competence and sociability were measured with 1 item.

One-sample *t*-tests were conducted (Bonferroni-corrected for 280 comparisons, *p* = 0.00018), comparing against the scale mid-point of 4 to choose the scenarios highly rated in the relevant concepts. Out of the 40 scenarios (see Appendix A), we selected 11 (see Table 2).

For the autonomy violations, we found two immoral scenarios rated significantly above the midpoint in violating autonomy and immorality, with one of these also being weird (Scenario 11) and the other rated as not weird (Scenario 4).

For the purity violations, we found four immoral scenarios focused on sex and food (the topics which have been argued are most associated with impurity [44]). The weird sex (Scenario 11) and food (Scenario 6) scenarios were both rated significantly above the midpoint of the scale as weird, immoral, and impure and were, as expected, considered physically disgusting. However, they were also rated as violating autonomy, which shows less distinction than expected for impurity. We were also able to find two non-weird moral violations related to food (Scenario 5) and sex (Scenario 17) that were rated as both immoral and not weird but also not impure and disgusting. This does, however, fit with the previous literature suggesting the associated disgust reaction to impurity is related to sex [79,80] or weirdness [75], not immorality.

We also chose appropriate comparison scenarios: a sexual scenario that was not significantly highly rated on any of the negative variables (Scenario 29), a scenario only rated as incompetent (Scenario 33), a scenario only rated as unsociable (Scenario 39), and a scenario that was only considered weird (Scenario 22). However, as none of the disgust scenarios were significantly high in disgust, and only two scenarios showed high disgust (Scenario 25 and Scenario 28), to allow inclusion of this comparison scenario, we chose the scenario framed as a behaviour they are enacting (clearing your throat, Scenario 28) rather than something that has more passively happened to them (eating a worm, Scenario 25), to fit better with the other scenarios. The chosen scenario (28) would have been significantly above the midpoint in disgust if not adjusting for multiple comparisons (*p* = 0.011).

## 6. Study 2b

In Study 2b, we use the scenarios chosen from the survey and ask participants which faces would be most likely to engage in these behaviours. We hypothesise that faces that are low morality, male, and low dominance will be considered more likely to engage in weird, impure scenarios: Immoral Purity, Sex Weird and Immoral Purity Food Weird. To investigate whether this reaction is specific to the “weirdness” of impurity, we also include Immoral Purity Food Nonweird and Immoral Purity Sex Nonweird to investigate whether these are also associated with immoral, low dominance, and male faces. Similarly, the nonmoral scenarios—Nonmoral Weird, Nonmoral Sex Nonweird, and Nonmoral Disgust Nonweird—contain elements of these impurity scenarios and, as such, may also be associated with low dominance faces and males, but not facial immorality. We also expect that those who have low morality and high dominance will be seen as more likely to engage in the following two scenarios: Immoral Autonomy Nonweird and Immoral Autonomy. Finally, based on the three-factor model of face perception, we also hypothesise that faces low in competence will be seen as more likely to engage in the Nonmoral Incompetent Nonweird scenario, and faces low in sociability will be considered more likely to engage in the Nonmoral Unsociable Nonweird scenario.

### 6.1. Method

#### 6.1.1. Sample Size

As per Study 1, we aimed for a minimum sample size of 16 to ideally 25 participants per social judgement to yield acceptable reliability [1,2,3]. We collected 299 participants; out of the initial sample, 9 entries were excluded for failing an image check (*n* = 1) or having zero variance in their data (*n* = 8), resulting in a final sample of 290 participants.

#### 6.1.2. Participants

The final sample of 290 participants (146 females, 142 males, 2 preferred not to say) was recruited through Prolific Academic; each was paid £2.15 for completing the study. Ages ranged from 18 to 73 (M_age_ = 36.96, SD_age_ = 12.19). Of the 290 participants, 266 participants self-reported English as their native language.

#### 6.1.3. Design and Materials

Study 2b used the same stimuli and design as Study 1: participants were asked to judge all stimuli on a single scenario (see Table 3 for the selected scenarios), resulting in 11 conditions. The between-subjects design is preferred to avoid carry-over effects [68,69].

#### 6.1.4. Procedure

The procedure was identical to Study 1, apart from the disgust and anger ratings, which were not collected in this study, and the inclusion of an image visibility check prior to the main photo rating task. Participants provided demographic information on their gender, age, ethnicity, and native language. As with Study 1, participants in the eleven scenario conditions can be seen in Table 3. They were asked to rate “How likely do you believe the person in the image is to engage in the following scenario:” with the photo and response scale directly below. Responses were made using the keyboard on a Likert scale from 1—Not at all to 9—Extremely.

The study was run using the software package Gorilla, build 2024-07-05 [82]. Participants could complete the study on any personal device with a keyboard (due to the response scale) and in their own time.

### 6.2. Results

#### 6.2.1. Data Preparation

Data preparation followed the protocol of Study 1.

#### 6.2.2. Internal Reliability

Cronbach’s Alpha was used to assess the internal reliability of the scenario ratings. Reliability was acceptable overall (i.e., Cronbach’s alpha was above 0.65; see Table S1–2b), with two exceptions: the Nonmoral Unsociable Nonweird scenario (α = 0.28) and the Immoral Autonomy Nonweird scenario (α = 0.56). Since both scenarios have low reliability, we have decided to exclude these from any further analyses, following our exclusion strategy from Study 1.

#### 6.2.3. Mixed Effects Models

The scenario ratings data, participant-level data and social trait data collected in Study 1 were collated and analysed to assess the impact of the face-based traits (competence, morality, sociability, dominance and attractiveness), demographic characteristics of the face (age, gender and ethnicity; collected from Face Research Lab London Set [67]) and participant demographic characteristics (age and gender) on to the criterion variables (i.e., ratings of the likelihood of engaging into each of the scenarios).

Face-level variables were standardised at the level of the face, and participant-level variables (i.e., participant demographic characteristics) were standardised at the participant level for each scenario.

Using lme4 [74] and Satterthwaite’s approximation (lmerTest [83]), we analysed the data with a mixed effects model including fixed effects of the face’s age, gender, ethnicity, attractiveness, competence, sociability, morality and dominance, and the participant’s age and gender, and the following random effects: random intercepts for each participant and each face, as well as random uncorrelated slopes for the by-age, by-gender, by-ethnicity, by-attractiveness, by-competence, by-sociability, by-morality and by-dominance effect of the participant, and for the by-participant age and by-participant gender effect of face. This model was fitted for each of the 9 scenario ratings with the “bobyqa” optimiser (Tables S2–S18).

**Figure 4:**
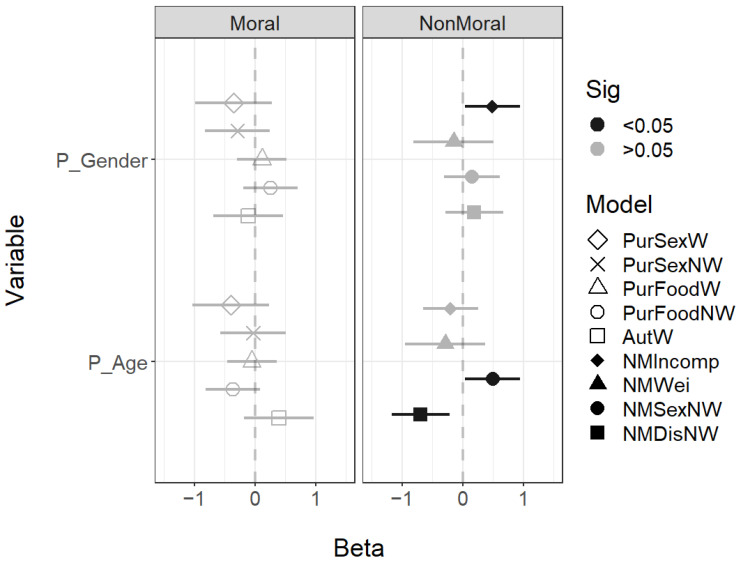
Regression coefficients for the participant variables. All predictors were standardised. Error bars show 95% confidence intervals; coefficients with CIs that exclude zero are highlighted in black. P_Age, participant age; and P_Gender, participant gender (1 = female). See Table 3 for descriptions of the scenarios linked to the model titles.

First, the participant’s own demographics only impacted three of the five nonmoral scenarios: Nonmoral Sex Nonweird, Nonmoral Disgust Nonweird, and Nonmoral Incompetence Nonweird. Older participants judge all faces as being more likely to engage in the Nonmoral Sexual Nonweird scenario, and younger participants judge all faces as being more likely to engage in the Nonmoral Disgust Nonweird scenario. Female participants also were more likely to judge a face as engaging in the Nonmoral Incompetence Nonweird scenario (Figure 4).

**Figure 5:**
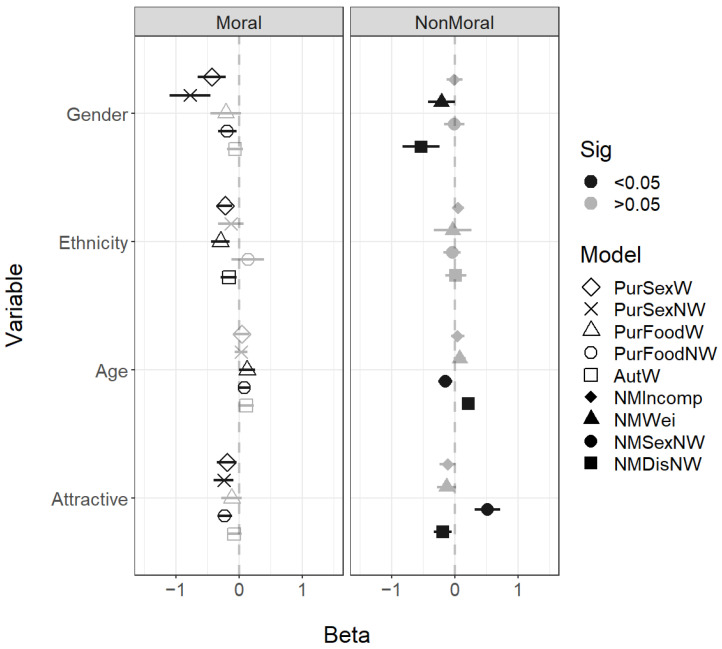
Regression coefficients for the face characteristic variables (Gender, Female = 1; Ethnicity, White = 1). All predictors were standardised. Error bars show 95% confidence intervals; coefficients with CIs that exclude zero are highlighted in black. See Table 3 for descriptions of the scenarios linked to the model titles.

For the facial characteristics of gender, ethnicity, age, and attractiveness, there was a different pattern of results for the moral and non-moral scenarios. For gender, most of the purity scenarios were again associated with male faces—specifically Immoral Purity Sex Weird, Immoral Purity Sex Nonweird, and Purity Food Nonweird. The Nonmoral Weird and Nonmoral Disgust Nonweird scenarios were also associated with male faces. The autonomy violation included in the analysis (Immoral Autonomy Weird) was only associated with white ethnicity. The Immoral Purity Sex Weird and Immoral Purity Food Weird scenarios were both also associated with white ethnicity. No nonmoral scenarios were associated with ethnicity. For age, both food-based purity violations—Immoral Purity Food Weird and Immoral Purity Food Nonweird—were associated with older faces, as was the Nonmoral Disgust Nonweird scenario. The Nonmoral Sexual Nonweird scenario was associated with younger faces. All purity scenarios other than the Immoral Purity Food Weird scenario were associated with unattractive faces, and so was the Nonmoral Disgust Nonweird scenario. The Nonmoral Sexual Nonweird scenario was associated with more attractive faces (Figure 5).

**Figure 6:**
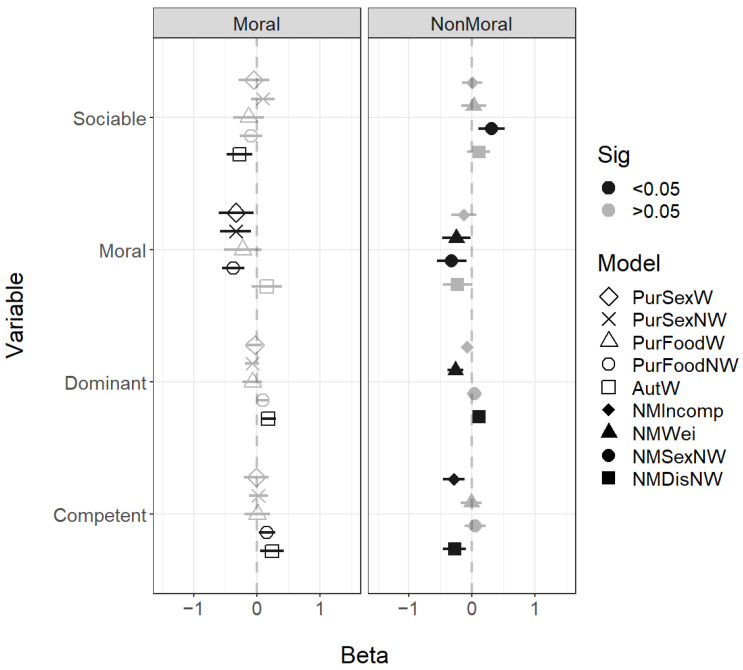
Regression coefficients for the face trait variables. All predictors were standardised. Error bars show 95% confidence intervals; coefficients with CIs that exclude zero are highlighted in black. See Table 3 for descriptions of the scenarios linked to the model titles.

For the facial social traits, lower sociability was associated with the Immoral Autonomy Weird scenario and higher sociability with the Nonmoral Sex Nonweird scenario. Lower facial morality was again associated with most of the immoral scenarios other than Immoral Purity, Food Weird, and Immoral Autonomy Weird. Interestingly, lower facial mortality was also associated with the nonmoral scenarios Nonmoral Weird and Nonmoral Sex Nonweird. In this study, dominance was not associated with most immoral scenarios other than Immoral Autonomy Weird, which was associated with higher dominance. Higher dominance was also associated with Nonmoral Disgust Nonweird and lower dominance with Nonmoral Weird. Higher competence was associated with two of the immoral scenarios: Immoral Purity Food Nonweird and Immoral Autonomy Weird. Lower competence was associated, as expected, with nonmoral incompetence and nonmoral disgust (Figure 6).

### 6.3. Discussion

Based on Study 1, we hypothesised that faces that are low morality, male, and low dominance would be considered more likely to engage in the weird, impure scenarios: Immoral Purity, Sex Weird and Immoral Purity Food Weird. Low morality and male faces were associated with Immoral Purity Sex Weird but not Immoral Purity Food Weird (which was only associated with white and older faces). Neither were associated with dominance. The nonweird comparison purity violations—Immoral Purity Food Nonweird and Immoral Purity Sex Nonweird—did somewhat support the hypothesised relationship, as both were associated with low morality and male faces. However, again, neither was associated with dominance. The nonmoral comparison scenarios—Nonmoral Weird, Nonmoral Sex Nonweird, and Nonmoral Disgust Nonweird—were predicted to not be associated with immorality, but both Nonmoral Weird and Nonmoral Sex Nonweird were associated with faces low in morality. Nonmoral Disgust Nonweird was not associated with facial morality. Nonmoral Disgust Nonweird and Nonmoral Weird were associated with males as predicted, but Nonmoral Sex Nonweird was not. Only Nonmoral Weird supported the predicted association with low dominance. Nonmoral Sex Nonweird was not associated with dominance and Nonmoral Disgust Nonweird was associated with high dominance. We predicted that Immoral Autonomy Weird would be associated with low morality and high dominance but only found an association with high dominance. Finally, as expected, we did find that faces low in competence were seen as more likely to engage in the Nonmoral Incompetent Nonweird scenario.

Additional, interesting non-hypothesised relationships emerged in this study. The demographic facial features (age and ethnicity), as well as facial traits of attractiveness, showed unexpected associations. Unattractive faces were rated as more likely to engage in most purity violations but also associated with clearing your throat in public (Nonmoral Disgust Nonweird). Attractive faces were only associated with having oral sex in a monogamous relationship (Nonmoral Sex Nonweird). Older faces were seen as more likely to engage in both purity food violations and autonomy violations and were more likely to clear their throat (Nonmoral Disgust Nonweird). Younger faces were associated with having oral sex in a monogamous relationship (Nonmoral Sex Nonweird). White faces were associated with all “weird” purity and autonomy violations.

The scenarios were also variably associated with the three-factor model. High competence and low sociability were associated with autonomy violation: a politician forcing identical appearance (Immoral Autonomy Weird). High competence was also associated with eating your daughter’s pet chicken (Immoral Purity Food Nonweird), whereas low competence was associated with clearing your throat in public (Nonmoral Disgust Nonweird) and being unable to perform in a new job (Nonmoral Incompetent Nonweird). Faces which appeared low in sociability were not associated with any scenario but faces high in sociability were associated with engaging in oral sex within a monogamous relationship (Nonmoral Sex Nonweird). Faces rated low in morality were also surprisingly associated with engaging in oral sex in a monogamous relationship (Nonmoral Sex Nonweird) and pretending to be a goblin (Nonmoral Weird).

## 7. General Discussion

In the above studies, we find different expectations about which facial traits were associated with moral violations, albeit with some patterns emerging. Our expectation based on the three-factor model of face perception was that all immoral scenarios would be associated with lower perceived facial morality (i.e., lower trustworthiness and honesty) [25]. In eight of the ten presented immoral scenarios across Study 1 and 2, this was the case. This aligns somewhat well with the three-factor model of social perception, which claims that competence, sociability, and morality are the core social traits humans use when forming first impressions of others [2,3,25], as these seemed to be used to judge the target’s likely intentions to commit certain immoral actions [25,53,84]. In line with prior suggestions that morality is deeply tied to cooperation and social cohesion [26,27], separate judgements that a person appears untrustworthy and dishonest did predict judgements they are more likely to also behave in a socially damaging manner, which could be beneficial in deciding whether we wish to approach a stranger or not [54,62,63,65,66,85,86,87]. However, this was not the case for all immoral actions. In Study 2, the autonomy violation, where a politician restricts freedoms, and the purity violation, where a scientist clones human cells for consumption, were not associated with low morality. Also interestingly, both the nonmoral sexual and weird scenarios from Study 2 were also associated with low facial morality. This suggests that, while this impression formation label of “morality” judgements may be associated with actual moral judgements, they may also more broadly apply to other social behaviours and not equally for all moral scenarios.

### 7.1. Facial Immorality and Immoral Behaviours

While not all immoral scenarios were associated with low-morality faces, some differences in the phrasing of the immoral scenarios may explain this result. For example, while similar in profession and action, the autonomy violation in Study 2, which involved a government official restricting freedoms, did differ from the autonomy violation in Study 1, which involved a corrupt politician. It may be that restricting freedoms—even harshly—is seen as part of a politician’s job, compared to corruption, which is not. The freedoms restricted were also “weird”, focused on restricting clothing choices, so not obviously beneficial to the politician as a sign of corruption. Therefore, it may be negative and immoral (as seen in Study 2) without meaning the person is dishonest or untrustworthy. The presence of additional information about the profession may affect how the scenario is judged. Thus, in the case of the authoritarian politician, the judgements are in line with the high competence judgements of politicians found in previous research [4,88]. Similarly, the cannibalism scenario in Study 1 was associated with low morality faces, but the cannibalism scenario committed by a scientist in Study 2 was not. Again, in this case, the judgements were more closely aligned to the judgements of scientists seen in other studies [2] of being older and white. This suggests that the social information about the professions of politicians and scientists, requiring levels of trustworthiness and honesty, may have predominated over the behaviour they are engaged in.

### 7.2. Competence and Sociability

It seems that the other social information about occupation and family present in the scenario affected those who were judged as likely to commit these violations. More broadly, as expected, it seems likely that competence and sociability were not tied specifically to the immorality of the actions but were associated with other social information contained in the scenarios. This fits with the idea that competence would be more associated with a stranger’s ability to carry out their intentions and sociability with a stranger’s support network in carrying out their intentions than the valence of the intention [25]. For example, it could be that the inclusion of specifically mentioning a daughter changed the associations of a person eating the family pet chicken (Study 2, Immoral Purity Food Nonweird) compared to the person eating their own pet dog (Study 1). Specifically, the similarities in the scenario may explain the associations with both scenarios to older males but mentioning that the person eating the chicken was a parent may have created additional associations with those who were competent but unsociable. Previous research suggests fathers are considered more competent than mothers [89] and older; traditional fathers are associated with unsociable behaviours such as being stern and authoritative [90] (rather than more modern fathers who are ascribed more maternal, warm traits [91]). As such, high competence with low sociability fits with the associations with a father eating his daughter’s pet chicken (Immoral Purity Food Nonweird, Study 2), likely due to the mention of seemingly authoritarian fatherhood [89]. This is similar to the effect on the judgements of politicians who restrict freedoms due to the mention of a competent but not sociable profession [4,88]. There were also other expected associations based on the social information contained in the scenario. In Study 2, the Nonmoral Incompetent Nonweird scenario of performing poorly at work was associated, as expected, with low competence faces. The finding that appearing low in competence is specifically associated with theft also fits with prior research, which shows that those who have been convicted of this crime are perceived as low in competence [92,93]. High sociability was only associated with giving a partner oral sex (Study 2, Nonmoral Sex Nonweird), fitting with more social competence linking to higher mating success [94]. This suggests that judgements of sociability and competence may be elicited non-specifically by both nonmoral and immoral behaviours.

### 7.3. Low Facial Morality and Nonmoral Behaviours

Interestingly, some nonmoral scenarios were also seen as being more likely committed by faces of “low morality”. Specifically, both the scenario showing oral sex in a happy relationship (Study 2, Nonmoral Nonweird Sex) and someone enjoying pretending to be a goblin (Study 2, Nonmoral Weird) were also associated with a facial appearance of low trustworthiness and honesty. This is despite these scenarios not being rated as immoral and negative in Study 2a. This suggests the mere presence of sexual or weird behaviours, regardless of the morality of the behaviour itself, may be associated with faces seen as low in trustworthiness and honesty. However, this still fits with these judgements being rooted in cooperation and social cohesion [26,27]. There are many reasons not to trust a person based on a behaviour that is not by itself immoral. There are adaptive reasons why someone behaving unpredictably or nonnormatively may impact your trust and willingness to cooperate with them, regardless of whether that behaviour is strictly immoral [95]. For example, behaving non-normatively is associated with selfishness, which has been found to cause avoidance in workplaces [96]. Similarly, the association between low morality and nonmoral sexual behaviour could be due to the nonreproductive act of oral sex being associated with unrestricted sociosexuality [97]. Unrestricted sociosexuality has associations with negative traits, such as dark triad traits [98], as well as infidelity, even within long-term relationships [99,100]. Oral sex has also been found as a specific dimension of sexual disgust—being one of only six dimensions within this concept—arguably meaning the potential for disease transmission relates to harsher judgements of those engaging in this act [101]. As such, it seems that behaviours that seem nonmoral but have connections to disgust or are commonly linked to other acts that can be considered immoral or selfish may also be associated with low-morality faces.

### 7.4. Male Sex and Impurity

Other than immorality, interestingly, the most consistent finding was that all but one of the purity violations and the physically disgusting behaviour were seen as more likely committed by male targets. This distinguished these scenarios from the other moral violations which were not linked to facial sex in Study 1 or Study 2. Sex differences are not unusual in face perception research, often mimicking traditional gender stereotypes [102]. It may, therefore, be unsurprising that immoral acts were associated with male faces, which are often ascribed to antisocial traits, such as low warmth, low emotionality, dishonesty, low cooperativeness, and poor quality as a parent [69,103]. It is also unsurprising that sexual violations would be stereotyped as male due to historically and cross-culturally sexually criminal behaviour being more often linked to male perpetrators [104,105] and more recently well-publicised concerns such as “incel” behaviours in men [106,107]. However, rather than broadly applying to all immoral scenarios due to these negative stereotypes or only to sexual impurity, the association seems to apply broadly to impurity—even non-sexual impurity—and physical disgust, providing further evidence of the similarity between reactions to physical disgust and impurity [40,41,42,47,50,70,108]. This finding could, therefore, also be linked to the common sex difference found in the disgust literature—where women are seen as more disgust-sensitive than men [109,110,111]. This could mean that men are considered more likely to engage in disgusting behaviours of all kinds because they are not as sensitive to these acts. In keeping with this, there is evidence of sex differences in pathogen avoidance behaviours such as handwashing [112,113,114]. Therefore, due to the strong connection between disgust and impurity, this could also increase perceptions that impurity is more likely to be committed by males who are less sensitive to the disgust associated with these acts.

### 7.5. Unattractiveness and Impurity

Unattractiveness was also associated with physically disgusting behaviour and impurity violations, although not as consistently as the male gender. Unlike Study 1, in Study 2, the sexually impure scenarios of necrophilia and masturbation by a disliked person were associated with unattractive faces, but so were the impure act of eating a pet chicken and the nonmoral disgust act of clearing your throat. Unattractiveness has long been associated with the moral domain due to the well-known halo effect associated with positive features such as attractiveness [115], with a bidirectional effect showing good behaviours increase attractiveness (and vice versa [116]). However, this was not a broad halo effect in this study. Only certain impure behaviours, especially sexual behaviours and physically disgusting behaviour, were associated with unattractiveness. Thus, in both our studies and previous research, unattractiveness is more associated specifically with violations of impurity [117], albeit in our studies, more specifically sexual impurity.

We also found that unattractiveness was associated with physical disgust. This fits with previous research suggesting that unattractiveness in humans, nonhumans, and objects is associated with both greater disease risk and disgust [118,119,120]. Like the broader increased sensitivity to disgust [109,110,111], prior research has also suggested that the association between disgust and unattractiveness is especially strong for women [121]. Because of this and the stronger association with sexually disgusting acts being more common in men [104,105,106,107], it is perhaps unsurprising that these acts were more associated with male faces. This is further supported by the more appealing, nonmoral sexual act in Study 2 being associated with more attractive faces and not specifically with male faces, suggesting it was not just that men were more associated with all sexual behaviours. As such, our findings support previous research correlating both impurity and physical disgust with unattractiveness but support a stronger association with sexual impurity than non-sexual.

### 7.6. Facial Dominance, Autonomy, and Impurity

In both Study 1 and Study 2, all three analysed autonomy violations were associated with high dominance: a politician restricting freedoms (Study 2), corruption (Study 1) and stealing (Study 1). As these violations are more directly harmful than purity violations, this pattern supports previous findings that high dominance is associated with aggression and risk-taking behaviours [122,123]. Previous research has also supported the connection between high dominance traits and criminality [92,124], specifically with corruption, as also seen in this study [125]. Dominance was associated with the powerful societal positions presented in the studies above: a corrupt politician (Study 1) and a politician with unusual authoritarian policies (Study 2, Immoral Autonomy Weird). However, the autonomy violation in Study 2 was not seen as more likely from those rated low in morality, suggesting this association is somewhat distinct from untrustworthiness. Highly dominant individuals often have high social status [126], and the appearance of dominance is often preferred in romantic partners [127], politicians [128], and colleagues [129]. As such, it may be that those who commit autonomy violations maintain some value as social connections (albeit with some necessary wariness and less stability [126]). As such, dominant individuals could offer benefits to close conspecifics [130,131], but the purity violations could not, thus likely motivating different social reactions.

However, not all scenarios showed this pattern with high dominance. The cannibalism following a plane crash parity violation in Study 1 and the disgust scenario of someone clearing their throat in public in Study 2 were also both associated with higher facial dominance. The cannibalism presented in Study 1 appeared to be an outlier as the only purity violation that was associated with high dominance. However, this was also the only act that required some form of violence and aggression towards another human and so could sensibly be associated with dominance [122,123]. This is different to the cannibalism scenario in Study 2, where a scientist clones human cells for consumption (Immoral Purity Food Weird), which is not associated with dominance. This could be due to scientists being usually seen as experts who gain social rank via prestige, as opposed to dominance, and are, therefore, more often trusted [132,133]. The act was also more clinical and less aggressive than the cannibalism in Study 1 and, therefore, would be less clearly linked to dominance [122,123]. Another outlier seems to be clearing your throat in public (Study 2, Nonmoral Nonweird Disgust), which is harder to explain via previous research. It could be that overtly displaying disgusting behaviour demonstrates a lack of pathogen concern, which has been associated with specific traits such as extroversion, openness, and sociosexuality [134]. In turn, there is some evidence that extroversion [135,136] and sociosexuality [137] are associated with dominance but not openness [136]. Future research could investigate whether overt displays of disgusting behaviours are considered dominant.

There is also some evidence that certain impure scenarios were associated with low dominance, perhaps due to the “weirdness” of these scenarios. Specifically, in Study 1, incest and eating a pet dog were also both seen as low dominance, as well as the “weird” scenario in Study 2. There is a motivation to conform to behaviours seen in high-status individuals, especially due to fear of negative evaluation by those who are dominant [138]. Bullying behaviour has been argued to be a process where aggressive, dominant individuals and conforming bystanders enforce social norms on individuals low in the social hierarchy [139]. Therefore, it is likely that those who do not conform—or are considered “weird”—as in the presented scenarios are low status and low dominance. As such, beyond nonmoral, nonnormative behaviour, the previously noted “weirdness” of some purity violations [37,75] could mean they are also assumed to be committed by dominant, low-status individuals.

### 7.7. Ethnicity and Age

Our findings regarding ethnicity and age seemed to follow some expected stereotypes about these demographic characteristics. Older faces were associated with some examples of impurity as well as physical disgust: the purity violations of a scientist cloning human meat for consumption (Study 2, Immoral Purity Food Weird), feeding a daughter her pet chicken (Study 2, Immoral Purity Food Nonweird), and eating your pet dog (Study 1), and also with the nonmoral scenario of clearing your throat in public (Study 2, Nonmoral Nonweird Disgust). Younger faces were only associated with oral sex in a long-term, happy relationship (Study 2, Nonmoral Nonweird Sex). However, this fits well with stereotypes and commonly seen characteristics of these positions. Being a strict, authoritarian father is associated with older men [91], politicians are expected to be older [140], scientists are stereotyped as older [2], eating dog meat is seen as more traditional [141], and clearing your throat becomes more difficult with age [142], and therefore these would be more likely associated with older generations. By contrast, younger faces being seen as more likely to engage in oral sex may rely on assumptions younger people are generally more sexually active [143]. As such, it seems that behaviours associated with traditional cultural and social attitudes were associated with older age. Similarly, stereotypes about professions and sexual behaviours also influenced judgements of those scenarios.

White faces were more associated with both scenarios focused on political positions (Study 1, Corruption and Study 2, Immoral Autonomy Weird), again likely reflecting stereotypes about who holds these positions. For example, in UK politicians, only 10% of the House of Commons were of a non-white ethnicity in 2019 [144]. The association between white ethnicity with incest (Study 1) and necrophilia (Study 2, Immoral Purity Sex Weird) does somewhat reflect the actual incidence of these crimes. For example, some population studies have found that abuse, including non-consensual incest, occurs at a comparatively high rate in white populations [145,146] and that those of white ethnicity are less likely to report sexual abuse and incest [147,148]. Similarly, necrophilia is a crime often associated with other extreme behaviours, such as homicide [149], which is frequently associated with white ethnicity due to famous cases [150]. As such, the results for ethnicity seemed to reflect stereotypes based on the most visible cases of criminal acts or the most commonly seen ethnicity for the stated profession.

## 8. Limitations and Future Directions

Due to the correlational nature of our work, we can only comment on potential relationships between facial characteristics and social judgements. Further experimental research should investigate the presence and potential impact of these associations. For example, a gap remains in whether the perceptions of low morality in both purity and autonomy violations lead to different social and behavioural responses. It is also unclear whether the characteristics associated with different forms of impurity (such as male gender, low dominance, and unattractiveness) also impact these responses. Previous research suggests that purity violations would be responded to with disgust and, therefore, avoidant behaviours [65,85,151], whereas autonomy violations would be responded to with anger and, therefore, aggressive approach behaviours [34,62,63,64]. Therefore, it could be investigated whether low-morality, unattractive, low-dominance, and male faces reacted to with greater avoidance than low-morality high-dominance faces who may be responded to with approach behaviours. As such, further research should investigate whether these combinations of facial traits would trigger different social responses.

There is evidence that negative evaluations in one domain, such as attractiveness [115,116,117], can cause negative evaluations in other areas, including morality. The three-factor approach to impression formation also highlights that negative morality evaluations tend to lead to global negative evaluations of competence and sociability [22]. It is, therefore, possible that some negative evaluations of the negative moral scenarios were related to global negative impressions. Further research could consider including a measure of global negative or positive impressions to investigate this further. However, in this study, we did find some positive associations, such as more competent appearances being associated with some immoral scenarios, suggesting there may not have been a global negative assessment for all scenarios.

Furthermore, many associations did differ greatly, even in response to very similar scenarios, which has important implications for the use of vignettes such as these in moral psychology research. Future research should, therefore, also investigate whether information contained within moral scenarios about professions and social relationships affects the judgement of these scenarios. Our results also suggest associations with traits such as attractiveness, dominance, and sex, which differ both between and within purity and autonomy scenarios. Based on the results associated with the nonmoral scenarios, these differences could be partly based on whether the scenario is sexual (low attractiveness), weird (low dominance), or disgusting (male gender/sex). However, currently, the results are correlational and based on a limited number of scenarios, so future research should consider how these aspects of purity violations impact those who are considered likely to engage in them.

## 9. Conclusions

Overall, this study provides novel insight into the social traits associated with the perceived likelihood of engaging in immoral behaviours. First, we provided evidence that morality is prioritised in face perception, with both purity and autonomy violations being associated with low-morality faces. However, low mortality was also associated with nonmoral sexual and weird behaviour, suggesting acts which are not immoral are also associated with faces low trustworthiness and honesty. The clearest distinction between purity and autonomy violations was found in facial sex and dominance: most purity violations (and physical disgust) were more associated with male sex, and autonomy violations were associated with ratings of high dominance. A similar finding for the physical disgust scenario suggests the male sex association could be due to the disgust present in the impurity scenarios. It also seems that both weirdness and sexual content in certain forms of impurity may lead to associations with low dominance (weirdness) and unattractiveness (sexual content). Our results suggest the morality dimension used in face perception research is linked to the perceived likelihood of engaging in both immoral behaviours and other socially relevant, nonmoral behaviours. Furthermore, other social judgements were not entirely homogonous within or between theory-based categories of moral violations. These findings indicate that increased complexity in future research on face perception and morality would be warranted.

## Figures and Tables

**Table 1:** Purity Violation and Autonomy Violations Adapted from Previous Research (see references).

Purity Violation Scenarios	
1	This person and their sibling waited for a time when nobody was around, and then they found a secret hiding place. Once they were hidden, this person and their sibling kissed each other on the mouth passionately [47].
2	This person’s plane crashed in the Himalayas. The only other survivor was a young boy. After a few days, the young boy died of his injuries. So, to survive, this person ate the boy [70].
3	This person’s pet dog was killed by a car in front of their house. This person had heard that some people occasionally eat dog meat, and they were curious what it tasted like. So, they cut up the body, cooked it and ate it for dinner [70].
**Autonomy Violation Scenarios**	
1	This person was driving a train. They realised if they did nothing, and the train remained on its current course it would kill five workmen. So, this person chose to change tracks, intentionally killing one workman instead of five [70].
2	This person is a politician who frequently gives speeches condemning corruption. But they are just trying to cover up the fact that they themselves will take bribes from the tobacco lobby to promote their legislation [71].
3	This person saw a woman with a guide dog sit down and place her handbag next to her. They realised she was blind and decided to steal her handbag. This person quietly took the handbag and left without the woman noticing [32].

**Table 2:** One-sample *t*-test coefficients and significance for chosen scenarios.

Category (Scenario #)	Immoral	Purity	Autonomy	Weird	Disgust	Incompetent	Unsociable
Immoral Purity Sex Weird (11)	28.97 *	12.79 *	9.77 *	19.36 *	14.12 *	−0.08	1.26
Immoral Purity Sex Nonweird (17)	5.11 *	0.78	−0.89	0.93	−4.71 *	−7.27 *	−2.14 *
Immoral Purity Food Weird (6)	13.10 *	4.75 *	8.55 *	23.38 *	6.33 *	−0.57	−1.32
Immoral Purity Food Nonweird (5)	5.70 *	2.00	−0.45	1.55	−1.76	−5.12 *	−2.28
Immoral Autonomy Weird (15)	13.83 *	2.01	6.77 *	6.70 *	−3.99	−1.37	1.30
Immoral Autonomy Nonweird (4)	9.04 *	−0.55	10.71 *	1.19	−5.16 *	0.36	0.75
Nonmoral Sex Nonweird (29)	−7.47 *	−6.56 *	−2.73	−10.49 *	−7.05 *	−2.95	−1.67
Nonmoral Disgust Nonweird (28)	1.19	−1.86	−1.87	−3.45	2.70	−4.04	1.28
Nonmoral Incompetent Nonweird (33)	0.20	−7.25 *	−2.29	−7.88 *	−10.30 *	8.42 *	−0.83 *
Nonmoral Unsociable Nonweird (39)	1.75	−10.8 *	−1.80	−4.54 *	−15.70 *	−4.44	5.37 *
Nonmoral Weird (22)	−0.98	−3.57	−3.29	8.84 *	−10.04 *	−6.73 *	−0.10

Note: * *p* < 0.00018, high scores in the variable bolded.

**Table 3:** Scenarios used in Study 2b.

Category (Variable Name)	Scenario
Immoral Purity Sex Weird (PurSexW)	This person is a morgue worker. They fantasise about what sex would be like with a corpse. One day, they act on this fantasy and have sex with a corpse.
Immoral Purity Sex Nonweird (PurSexNW)	This person knows their colleague does not like them and their colleague thinks they are unattractive. They leave them alone but have sexual fantasies and masturbate while thinking about this colleague.
Immoral Purity Food Weird (PurFoodW)	This person is a scientist who is working on a project to clone sheep cells for meat. They decide to clone some human cells to make a steak of human meat and eat this steak.
Immoral Purity Food Nonweird (PurFoodNW)	This person’s daughter had a chicken as a pet. They kill it, make a roast dinner out of it, and feed it to the family, including their daughter.
Immoral Autonomy Weird (AutW)	This person is a politician. They endorse policies which force their citizens into wearing identical clothing and having identical haircuts.
Immoral Autonomy Nonweird (AutNW)	This person is a government official. They endorse policies which prevent their citizens from accessing social media or choosing which newspapers they can read or which television programs they can watch.
Nonmoral Sex Nonweird (NMSexNW)	This person is in a long-term relationship. They choose to give their partner oral sex on a regular basis. They are both happy with this and feel it improves their sex life.
Nonmoral Disgust Nonweird (NMDisNW)	This person is recovering from a cold. They loudly clear their throat of mucus in public.
Nonmoral Incompetent Nonweird (NMIncomp)	This person has recently been hired in an entry-level position. Two weeks in, their line manager has raised concerns about their inability to grasp even the simplest of tasks
Nonmoral Unsociable Nonweird (NMUnsoc)	This person does not enjoy going out with their friends, even in small groups. They have rejected so many offers, their friends are considering not inviting them anymore.
Nonmoral Weird (NMWei)	This person pretends to be a goblin to relieve stress. They would usually walk in a crouched manner around their house putting things in a sack while making goblin noises.

## Data Availability

The anonymised data (following exclusions), information about the stimuli and data analysis R script are available at https://osf.io/mdxa7/.

## Supplementary Materials

The following supporting information can be downloaded at: https://www.mdpi.com/article/10.3390/bs14121205/s1, Table S1-1: Cronbach’s Alpha values for the eight facial traits, three purity and three non-purity violation scenarios in Study 1; Table S1-2b: Cronbach’s Alpha values for the eleven scenarios in Study 2b; Table S2: Correlations among the items forming each trait, and other face-level measures (* indicates *p* < 0.05); Table S3: Coefficients and *p*-values for the fixed effects of the predictors for the random intercept and slopes model, when using mixed-effects data to predict likelihood of engagement in each of the purity violation scenarios, for male and female faces; Table S4: Coefficients and *p*-values for the fixed effects of the predictors for the random intercept and slopes model, when using mixed-effects data to predict likelihood of engagement in each of the non-purity violation scenarios, for male and female faces; Table S5: Regression coefficients for the “Cannibalism” scenario (Study 1); Table S6: Regression coefficients for the “Corruption” scenario (Study 1); Table S7: Regression coefficients for the “Eating Dog Meat” scenario (Study 1); Table S8: Regression coefficients for the “Incest” scenario (Study 1); Table S9: Regression coefficients for the “Stealing” scenario (Study 1); Table S10: Regression coefficients for the “Immoral Autonomy Weird” scenario (Study 2b); Table S11: Regression coefficients for the “Nonmoral Disgust Nonweird” scenario (Study 2b); Table S12: Regression coefficients for the “Nonmoral Incompetent Nonweird” scenario (Study 2b); Table S13: Regression coefficients for the “Nonmoral Sex Nonweird” scenario (Study 2b); Table S14: Regression coefficients for the “Nonmoral Weird” scenario (Study 2b); Table S15: Regression coefficients for the “Immoral Purity Food Nonweird” scenario (Study 2b); Table S16: Regression coefficients for the “Immoral Purity Food Weird” scenario (Study 2b); Table S17: Regression coefficients for the “Immoral Purity Sex Nonweird” scenario (Study 2b); Table S18: Regression coefficients for the “Immoral Purity Sex Weird” scenario (Study 2b).

## Appendix A. Scenarios for Study 2a

1. This person is a politician who frequently gives speeches condemning corruption. But they are just trying to cover up the fact that they themselves will take bribes from the tobacco lobby to promote their legislation.
2. This person sees a woman with a guide dog sit down and place her handbag next to her. They realise she is blind and decide to steal her handbag. This person quietly took the handbag and left without the woman noticing.
3. This person is arguing with their partner. They suddenly raise their hand and slap their partner across the face.
4. This person is a government official. They endorse policies which prevent their citizens from accessing social media or choosing which newspapers they can read or which television programs they can watch.
5. This person’s daughter had a chicken as a pet. They kill it, make a roast dinner out of it, and feed it to the family, including their daughter.
6. This person is a scientist who is working on a project to clone sheep cells for meat. They decide to clone some human cells to make a steak of human meat and eat this steak.
7. This person’s pet dog is killed by a car in front of their house. This person had heard that some people occasionally eat dog meat, and they were curious about what it tasted like. So, they cut up the body, cook it and eat it for dinner.
8. This person’s partner was cooking for them. As the partners were preparing the food to cook, they cut themselves, and some blood dripped into the meal. This person noticed, but they ate it anyway.
9. This person and their sibling wait for a time when nobody is around, and then they find a secret hiding place. Once hidden, this person and their sibling kiss each other passionately on the mouth.
10. This person is a fertility doctor. From time to time, they choose to make embryos using their own DNA. They then artificially inseminate their patients with these instead of ones made with the patients’ preferred partners.
11. This person is a morgue worker. They fantasise about what sex would be like with a corpse. One day, they act on this fantasy and have sex with a corpse.
12. This person and their closest friend record when they have sex with their partners. They then secretly show these recordings to each other.
13. This person likes to go into public places and pretend to be a dog. To scare passers-by, they walk on all fours, growl and bark loudly.
14. This person is a government official. They use government money to fund their hobby of collecting doorknobs and teaspoons.
15. This person is a politician. They endorse policies that force their citizens to wear identical clothing and have identical haircuts.
16. This person is often invited to people’s houses. When they are there, they always steal exactly one fork and the person’s hairbrush.
17. This person knows their colleague does not like them, and their colleague thinks they are unattractive. They leave them alone but have sexual fantasies and masturbate while thinking about this colleague.
18. This person is in an elevator at work. The person in front of them smells nice. Sniffing the person in front of them gives them sexual gratification.
19. This person is looking for a romantic relationship. They exclusively choose to have sex with people who are unhygienic.
20. This person is looking for a romantic relationship. They exclusively choose to have sex with people who are uneducated and with few monetary resources.
21. This person hoards objects. They have a particular interest in removing the heads of every doll they have come across. They have 3023 doll heads displayed in their house.
22. This person pretends to be a goblin to relieve stress. They would usually walk in a crouched manner around their house, putting things in a sack and making goblin noises.
23. This person takes photos of their hand every time they shake a stranger’s hand. They then display these photos around their house.
24. This person has a deep fascination with cats. During the weekend, they often glue cat hair to themselves from head to toe, as this helps them feel like a cat.
25. This person bites into an apple, which has a small worm in it. They do not realise there is a worm in the apple until after they have swallowed it.
26. This person is visiting a clinic to undergo urine tests. Accidentally, when they screw the lid on, they spill some warm urine on their hand.
27. This person works at a hospital. A pregnant woman’s water breaks, and this person slips on this water and is covered in it.
28. This person is recovering from a cold. They loudly clear their throat of mucus in public.
29. This person is in a long-term relationship. They choose to give their partner oral sex on a regular basis. They are both happy with this and feel it improves their sex life.
30. This person is having a romantic evening with their partner. They decide to try anal sex for the first time. They take it slow and are both happy with how this goes.
31. This person is in a long-term relationship. For fun, they decide to try roleplay and dressing up while they have sex. They both enjoy this and decide to do it regularly.
32. This person is in a long-term relationship. They agree with their partner to try restraining them and using a whip. They agree on a safe word in advance and are both happy to try this.
33. This person has recently been hired in an entry-level position. Two weeks in, their line manager has raised concerns about their inability to grasp even the simplest of tasks.
34. This person has been consistently performing poorly in an educational course, with their grades hovering around the bottom 10%. In their latest exams, they are unable to answer most questions despite studying beforehand, as their problem-solving skills are extremely inefficient.
35. This person has decided to pursue their dream of going to culinary school. Despite being enrolled for 6 months, they still find themselves incapable of remembering the basics or following simple instructions.
36. This person has been joining their group of friends and going to escape rooms more regularly. They do not contribute much to the puzzle, as they often cannot decipher the clues or make the relevant connections.
37. This person does not have difficulties making friends but does not enjoy spending time with them, choosing not to put effort into the friendships. Eventually, these friendships fizzle out.
38. This person does not make an effort to help or support any of their friends when their friends seem like they need it. Their friends are there for them and gladly support them as best they can.
39. This person does not enjoy going out with their friends, even in small groups. They have rejected so many offers their friends are considering not inviting them anymore.
40. This person describes themself as a “lone wolf”. They keep to themselves in social situations and make no effort to get to know other people.
