# Supplementary material for: The Relationship Between Face-Based First Impressions and Perceptions of Purity and Compared to Other Moral Violations
Source: Behav Sci (Basel). 2024 Dec 16;14(12):1205. doi: 10.3390/bs14121205 (PMC11673250; doi:10.3390/bs14121205)
Supplement: Supplementary file 1 [file behavsci-14-01205-s001.zip › behavsci-3337611-supplementary.pdf]

APPENDIX: SUPPLEMENTARY INFORMATION

Consistency of measures

Tables S1-1 and S1-2b show Cronbach's Alpha (non-robust) values for the measures used in Study 1 (Table S1-1) and Study 2b (S1-2b).

Table S1-1
Cronbach's Alpha values for the eight facial traits, three purity and three non-purity violation scenarios in Study 1.

Judgement	Cronbach's Alpha
Competence	.80
Capability	.83
Friendliness	.89
Likeability	.79
Honesty	.75
Trustworthiness	.82
Dominance	.85
Masculinity	.99
Purity violation scenario: "incest"	.81
Purity violation scenario: "cannibalism"	.89
Purity violation scenario: "eating dog meat"	.78
Non-purity violation scenario: "train"	.22
Non-purity violation scenario: "corruption"	.63
Non-purity violation scenario: "stealing"	.78

Table S1-2b
Cronbach's Alpha values for the eleven scenarios in Study 2b.

Judgement	Cronbach's Alpha
Immoral Autonomy Nonweird	.56
Immoral Autonomy Weird	.66
Nonmoral Disgust Nonweird	.91
Nonmoral Incompetent Nonweird	.77
Nonmoral Sex Nonweird	.82
Nonmoral Unsociable Nonweird	.28
Nonmoral Weird	.68
Immoral Purity Food Nonweird	.85
Immoral Purity Food Weird	.74
Immoral Purity Sex Nonweird	.90
Immoral Purity Sex Weird	.88

Confirmatory Factor Analysis

The correlations between the trait ratings are shown in Table S2, and are consistent with both the three-factor structure we were expecting (competence, morality and sociability) and the two-factor structure (competence and warmth), with dominance and masculinity as separate factors.

Table S2

Correlations among the items forming each trait, and other face-level measures (* indicates p <.05).

	Capab.	Friend.	Likeab.	Honesty	Trust.	Domin.	Masc.	Age	Attract.	Gender	Ethnicity
Competence	.878*	.572*	.727*	.601*	.752*	-.038	-.481*	-.034	.573*	.483*	.269*
Capability		.640*	.745*	.594*	.758*	.047	-.519*	.045	.570*	.529*	.219*
Friendliness			.760*	.667*	.737*	-.269*	-.330*	-.145	.459*	.301*	.114
Likeability				.723*	.838*	-.221*	-.552*	-.199*	.591*	.535*	.126
Honesty					.786*	-.349*	-.556*	-.224*	.581*	.508*	-.215*
Trustworthiness						-.319*	-.656*	-.185	.617*	.630*	.033
Dominance							.339*	.458*	-.081	-.255*	.050
Masculinity								.141	-.489*	-.982*	.087
Age									-.381*	-.061	-.040
Attractiveness										.452*	-.199*
Gender											-.078

To establish which factor structure is the most suitable for our dataset, we compared 4 models: Model 0, where competence and capability loaded onto Competence, friendliness and likeability loaded onto Sociability, trustworthiness and honesty loaded onto Morality while dominance and masculinity remained their own factors; Model 1, where Competence, Sociability and Morality followed Model 0, but dominance and masculinity loaded onto Power; Model 2 where Competence and Power followed Model 1 but friendliness, likeability, trustworthiness and honest loaded onto Warmth; and Model 3 where Warmth followed Model 2 but competence, capability, dominance and masculinity loaded onto Competence/Power. Within the analysis, each item was loaded only onto its hypothesized factor, and the latent variables were allowed to correlate with each other. We used the lavaan package for R (Rosseel, 2012) to conduct the CFA.

Model 0 was the best fit, based on global indices of fit ($SRMR_{Model0}$ = .033, $RMSEA_{Model0}$ = .123, CFI_{Model0} = .973, TLI_{Model0} = .936), followed by Model 1 ($SRMR_{Model1}$ = .071, $RMSEA_{Model1}$ = .175, CFI_{Model1} = .936, TLI_{Model1} = .872), Model 2 ($SRMR_{Model2}$ = .079, $RMSEA_{Model2}$ = .179, CFI_{Model2} = .918, TLI_{Model2} = .866) and Model 3 ($SRMR_{Model3}$ = .112, $RMSEA_{Model3}$ = .206, CFI_{Model3} = .879, TLI_{Model3} = .822).

This difference was supported by chi-square tests for the difference in model fit, where Model 2 fit better than Model 3 (χ^2 diff(2) = 28.86, $p < .001$), Model 1 fit better than Model 2 (χ^2 diff(2) = 14.97, $p = .002$) and Model 0 fit better than Model 1: χ^2 diff(2) = 27.15, $p < .001$.

Multicollinearity issues

The decision to not include masculinity as a fixed effect in the mixed-effects analysis was made after running the mixed effects model separately for the male and the female faces; the analyses detailed below indicated there was no difference in masculinity depending on the face gender (Tables S3 and S4). As such, we decided to include face gender but not face masculinity in subsequent analyses.

Face-level variables were standardised at the level of the face, and participant-level variables (i.e., participant demographic characteristics) were standardised at participant-level, for each scenario.

Using lme4 and Satterthwaite's approximation, we analysed the data with a mixed effects model including fixed effects of the face's age, ethnicity, attractiveness, competence, sociability, morality, dominance and masculinity, and the participant's age, gender, anger and disgust, and the following random effects: random intercepts for each participant and each face, as well as random uncorrelated slopes for the by-age, by-ethnicity, by-attractiveness, by-discipline, by-competence, by-sociability, by-morality, by-dominance and by-masculinity effect of participant, and for the by-participant age, by-participant gender, by-participant anger and by-participant disgust effect of face. This model was fitted separately for male faces and female faces, and separately for each of the three purity and two non-purity scenario ratings, with the "bobyqa" optimizer.

Results indicated there was no difference in masculinity depending on the face gender (Tables S3 and S4).

Table S3
Coefficients and p-values for the fixed effects of the predictors for the random intercept and slopes model, when using mixed-effects data to predict likelihood of engagement in each of the purity violation scenarios, for male and female faces.

**Predictor**	**Male Faces**			**Female Faces**		
	**Incest B *(p)***	**Cannibalism B *(p)***	**Eating Dog Meat B *(p)***	**Incest B *(p)***	**Cannibalism B *(p)***	**Eating Dog Meat B *(p)***
Intercept	3.866 (<.001)	5.713 (<.001)	3.312 (<.001)	2.978 (<.001)	4.240 (<.001)	2.405 (<.001)
Face Age	0.017 (.885)	0.128 (.205)	0.221 (.023)	-0.062 (.451)	0.014 (.854)	0.146 (.025)
Face Ethnicity (Non-White)	-0.443 (<.001)	-0.082 (.490)	0.177 (.155)	-0.323 (.005)	-0.009 (.919)	0.236 (.079)
Attractiveness	-0.274 (.012)	-0.160 (.057)	-0.157 (.101)	-0.119 (.379)	-0.012 (.906)	0.034 (.714)
Competence	-0.060 (.683)	-0.013 (.915)	-0.016 (.902)	-0.145 (.297)	-0.123 (.206)	0.043 (.634)
Sociability	0.117 (.393)	-0.071 (.570)	0.164 (.238)	-0.209 (.194)	0.115 (.395)	0.181 (.147)
Morality	-0.491 (.009)	-0.277 (.103)	-0.535 (.003)	0.083 (.620)	-0.273 (.067)	-0.310 (.020)
Dominance	-0.289 (.011)	0.046 (.650)	-0.268 (.019)	-0.153 (.049)	0.148 (.033)	-0.003 (.957)
Masculinity	0.011 (.924)	0.059 (.568)	-0.059 (.555)	-0.030 (.770)	0.067 (.466)	0.140 (.101)
Participant Age	0.250 (.494)	0.051 (.875)	0.120 (.781)	0.477 (.121)	0.138 (.633)	0.003 (.991)
Participant Gender (Female)	0.099 (.771)	-0.384 (.244)	-0.018 (.967)	0.131 (.638)	-0.361 (.215)	-0.127 (.665)
Participant Anger	0.512 (.233)	0.065 (.858)	0.882 (.111)	0.718 (.049)	0.306 (.342)	0.475 (.200)

Participant Disgust	-0.013 (.974)	-0.319 (.411)	-0.252 (.636)	-0.271 (.414)	-0.791 (.028)	-0.039 (.913)

Table S4

Coefficients and p-values for the fixed effects of the predictors for the random intercept and slopes model, when using mixed-effects data to predict likelihood of engagement in each of the non-purity violation scenarios, for male and female faces.

**Predictor**	**Male Faces**		**Female Faces**	
	**Corruption B *(p)***	**Stealing B *(p)***	**Corruption B *(p)***	**Stealing B *(p)***
Intercept	4.462 (<.001)	4.247 (<.001)	3.792 (<.001)	3.213 (<.001)
Face Age	-0.026 (.745)	-0.022 (.850)	0.091 (.307)	-0.046 (.453)
Face Ethnicity (Non-White)	-0.197 (.024)	0.146 (.195)	-0.083 (.445)	0.002 (.984)
Attractiveness	-0.230 (.015)	-0.059 (.579)	-0.031 (.790)	0.079 (.380)
Competence	0.146 (.170)	-0.236 (.127)	0.027 (.806)	-0.091 (.282)
Sociability	0.093 (.410)	0.197 (.287)	-0.099 (.571)	-0.047 (.702)
Morality	-0.364 (.047)	-0.451 (.045)	-0.120 (.456)	-0.176 (.251)
Dominance	0.157 (.103)	0.098 (.486)	0.158 (.036)	0.167 (.025)
Masculinity	0.154 (.124)	0.255 (.065)	0.023 (.880)	0.089 (.226)
Participant Age	-0.282 (.313)	0.490 (.182)	-0.103 (.712)	0.803 (.032)
Participant Gender	0.260 (.411)	-0.953 (.017)	-0.004 (.990)	-1.419 (.001)

(Female)				
Participant Anger	1.090 (.031)	0.815 (.121)	0.736 (.137)	0.804 (.115)
Participant Disgust	-0.386 (.381)	0.091 (.859)	-0.225 (.612)	-0.041 (.934)

Mixed effects models

Tables S5-S9 (Study 1) and S10-S18 (Study 2b) give the numeric values of the regression coefficients plotted in the main text, along with 95% CIs and p-values based on Satterthwaite-adjusted degrees of freedom. Unless otherwise noted, all predictors were standardized (zscored), across participants for participant-level variables and across faces for face-level variables.

Table S5

Regression coefficients for the "Cannibalism" scenario (Study 1).

Predictor	*B*	CI_{low}	CI_{high}	*p*
Age	0.071	-0.063	0.205	0.303
Ethnicity	-0.080	-0.234	0.075	0.316
Gender	-0.417	-0.619	-0.216	<.001
Attractive	-0.129	-0.260	0.001	0.057
Competent	-0.023	-0.182	0.136	0.778
Sociable	0.031	-0.164	0.225	0.759
Moral	-0.379	-0.633	-0.125	0.004
Dominant	0.109	0.005	0.212	0.044
P_Age	0.092	-0.465	0.650	0.749
P_Gender	-0.375	-0.927	0.177	0.198
P_Anger	0.182	-0.434	0.798	0.569
P_Disgust	-0.545	-1.202	0.111	0.119

Table S6

Regression coefficients for the "Corruption" scenario (Study 1).

Predictor	*B*	CI_{low}	CI_{high}	*p*
Age	0.073	-0.026	0.172	0.152
Ethnicity	-0.156	-0.291	-0.020	0.032
Gender	-0.068	-0.219	0.084	0.387
Attractive	-0.134	-0.349	0.080	0.229
Competent	0.097	-0.071	0.265	0.266

Sociable	0.021	-0.217	0.259	0.864
Moral	-0.332	-0.609	-0.054	0.024
Dominant	0.210	0.091	0.330	0.002
P_Age	-0.196	-0.683	0.290	0.439
P_Gender	0.132	-0.421	0.686	0.645
P_Anger	0.922	0.086	1.757	0.044
P_Disgust	-0.309	-1.079	0.461	0.442

Table S7

Regression coefficients for the “Eating Dog Meat” scenario (Study 1).

**Predictor**	***B***	**CI_{low}**	**CI_{high}**	***p***
Age	0.162	0.046	0.278	0.009
Ethnicity	0.189	-0.027	0.405	0.098
Gender	-0.234	-0.409	-0.058	0.014
Attractive	-0.115	-0.276	0.045	0.167
Competent	0.001	-0.203	0.206	0.989
Sociable	0.177	-0.038	0.393	0.110
Moral	-0.494	-0.754	-0.233	<.001
Dominant	-0.177	-0.313	-0.040	0.015
P_Age	0.065	-0.599	0.729	0.851
P_Gender	-0.070	-0.733	0.592	0.838
P_Anger	0.688	-0.132	1.507	0.120
P_Disgust	-0.150	-0.970	0.670	0.725

Table S8

Regression coefficients for the “Incest” scenario (Study 1).

**Predictor**	***B***	**CI_{low}**	**CI_{high}**	***p***
Age	0.0001	-0.146	0.145	0.998
Ethnicity	-0.421	-0.587	-0.254	0.001

Gender	-0.201	-0.335	-0.067	0.005
Attractive	-0.178	-0.379	0.023	0.091
Competent	-0.121	-0.348	0.107	0.303
Sociable	0.001	-0.246	0.249	0.991
Moral	-0.296	-0.577	-0.014	0.043
Dominant	-0.209	-0.340	-0.079	0.003
P_Age	0.358	-0.265	0.982	0.275
P_Gender	0.114	-0.470	0.698	0.706
P_Anger	0.609	-0.114	1.333	0.116
P_Disgust	-0.137	-0.825	0.552	0.702

Table S9

Regression coefficients for the "Stealing" scenario (Study 1).

**Predictor**	***B***	**CI_{low}**	**CI_{high}**	***p***
Age	-0.004	-0.128	0.120	0.949
Ethnicity	0.056	-0.113	0.225	0.519
Gender	-0.120	-0.295	0.055	0.187
Attractive	0.021	-0.124	0.166	0.776
Competent	-0.194	-0.379	-0.008	0.044
Sociable	0.162	-0.136	0.460	0.296
Moral	-0.466	-0.823	-0.109	0.015
Dominant	0.231	0.085	0.377	0.004
P_Age	0.640	-0.006	1.286	0.071
P_Gender	-1.176	-1.834	-0.518	0.003
P_Anger	0.805	-0.110	1.720	0.105
P_Disgust	0.032	-0.900	0.963	0.948

Table S10

Regression coefficients for the "Immoral Autonomy Weird" scenario (Study 2b).

Predictor	*B*	CI_{low}	CI_{high}	*p*
Age	0.116	-0.004	0.236	0.065
Ethnicity	-0.162	-0.287	-0.037	0.014
Gender	-0.063	-0.186	0.061	0.324
Attractive	-0.073	-0.191	0.044	0.226
Competent	0.241	0.054	0.428	0.014
Sociable	-0.279	-0.486	-0.072	0.010
Moral	0.152	-0.089	0.392	0.220
Dominant	0.178	0.059	0.296	0.005
P_Age	0.395	-0.178	0.968	0.188
P_Gender	-0.112	-0.686	0.461	0.704

Table S11
Regression coefficients for the “Nonmoral Disgust Nonweird” scenario (Study 2b).

Predictor	*B*	CI_{low}	CI_{high}	*p*
Age	0.212	0.117	0.308	<.001
Ethnicity	0.017	-0.150	0.184	0.842
Gender	-0.535	-0.828	-0.242	0.001
Attractive	-0.185	-0.326	-0.045	0.012
Competent	-0.278	-0.459	-0.096	0.004
Sociable	0.112	-0.070	0.295	0.230
Moral	-0.227	-0.467	0.012	0.066
Dominant	0.117	0.014	0.219	0.030
P_Age	-0.694	-1.174	-0.215	0.009
P_Gender	0.191	-0.287	0.668	0.442

Table S12
Regression coefficients for the “Nonmoral Incompetent Nonweird” scenario (Study 2b).

Predictor	*B*	CI_{low}	CI_{high}	*p*

Age	0.046	-0.058	0.151	0.392
Ethnicity	0.055	-0.034	0.144	0.232
Gender	-0.007	-0.133	0.120	0.920
Attractive	-0.112	-0.238	0.014	0.089
Competent	-0.289	-0.459	-0.118	0.002
Sociable	0.005	-0.154	0.163	0.955
Moral	-0.127	-0.329	0.075	0.223
Dominant	-0.079	-0.160	0.002	0.062
P_Age	-0.200	-0.655	0.255	0.399
P_Gender	0.490	0.036	0.944	0.045

Table S13
Regression coefficients for the "Nonmoral Sex Nonweird" scenario (Study 2b).

**Predictor**	***B***	**CI_{low}**	**CI_{high}**	***p***
Age	-0.145	-0.255	-0.035	0.013
Ethnicity	-0.040	-0.174	0.093	0.553
Gender	-0.007	-0.166	0.152	0.936
Attractive	0.517	0.313	0.720	<.001
Competent	0.047	-0.121	0.215	0.584
Sociable	0.315	0.105	0.525	0.004
Moral	-0.321	-0.557	-0.085	0.009
Dominant	0.043	-0.071	0.158	0.462
P_Age	0.492	0.037	0.948	0.045
P_Gender	0.153	-0.303	0.608	0.517

Table S14
Regression coefficients for the "Nonmoral Weird" scenario (Study 2b).

**Predictor**	***B***	**CI_{low}**	**CI_{high}**	***p***
Age	0.078	-0.022	0.178	0.132

Ethnicity	-0.035	-0.333	0.263	0.819
Gender	-0.212	-0.424	-0.0004	0.059
Attractive	-0.129	-0.281	0.022	0.102
Competent	-0.010	-0.178	0.157	0.904
Sociable	0.026	-0.173	0.225	0.797
Moral	-0.249	-0.477	-0.021	0.035
Dominant	-0.263	-0.392	-0.134	<.001
P_Age	-0.286	-0.945	0.374	0.405
P_Gender	-0.150	-0.809	0.509	0.660

Table S15

Regression coefficients for the “Immoral Purity Food Nonweird” scenario (Study 2b).

**Predictor**	***B***	**CI_{low}**	**CI_{high}**	***p***
Age	0.084	0.004	0.165	0.045
Ethnicity	0.140	-0.120	0.399	0.300
Gender	-0.184	-0.329	-0.039	0.018
Attractive	-0.225	-0.338	-0.112	<.001
Competent	0.159	0.026	0.292	0.023
Sociable	-0.097	-0.273	0.078	0.281
Moral	-0.375	-0.555	-0.196	<.001
Dominant	0.095	-0.007	0.197	0.074
P_Age	-0.365	-0.814	0.085	0.124
P_Gender	0.254	-0.195	0.704	0.278

Table S16

Regression coefficients for the “Immoral Purity Food Weird” scenario (Study 2b).

**Predictor**	***B***	**CI_{low}**	**CI_{high}**	***p***
Age	0.128	0.004	0.252	0.046
Ethnicity	-0.294	-0.436	-0.151	<.001

Gender	-0.209	-0.454	0.036	0.105
Attractive	-0.117	-0.281	0.048	0.169
Competent	0.005	-0.202	0.212	0.961
Sociable	-0.133	-0.376	0.110	0.288
Moral	-0.230	-0.527	0.067	0.134
Dominant	-0.071	-0.228	0.085	0.375
P_Age	-0.054	-0.461	0.354	0.799
P_Gender	0.115	-0.293	0.523	0.586

Table S17
Regression coefficients for the "Immoral Purity Sex Nonweird" scenario (Study 2b).

**Predictor**	***B***	**CI_{low}**	**CI_{high}**	***p***
Age	0.032	-0.072	0.135	0.551
Ethnicity	-0.130	-0.333	0.074	0.221
Gender	-0.771	-1.097	-0.446	<.001
Attractive	-0.240	-0.397	-0.084	0.004
Competent	0.023	-0.132	0.178	0.773
Sociable	0.092	-0.098	0.282	0.346
Moral	-0.340	-0.587	-0.092	0.009
Dominant	-0.079	-0.191	0.032	0.171
P_Age	-0.030	-0.568	0.509	0.915
P_Gender	-0.289	-0.828	0.249	0.303

Table S18
Regression coefficients for the "Immoral Purity Sex Weird" scenario (Study 2b).

**Predictor**	***B***	**CI_{low}**	**CI_{high}**	***p***
Age	0.042	-0.080	0.163	0.503
Ethnicity	-0.222	-0.338	-0.106	<.001
Gender	-0.433	-0.657	-0.210	0.001

Attractive	-0.191	-0.347	-0.036	0.018
Competent	-0.012	-0.209	0.185	0.906
Sociable	-0.053	-0.298	0.192	0.675
Moral	-0.332	-0.610	-0.055	0.022
Dominant	-0.038	-0.157	0.080	0.527
P_Age	-0.399	-1.032	0.235	0.231
P_Gender	-0.350	-0.984	0.283	0.290
